# Testing the Preliminary Validity of a Multidimensional Framework for Studying the Effects of Cancer Health Literacy on Cancer Screening Behaviors among Diverse Populations

**DOI:** 10.3390/ijerph17092987

**Published:** 2020-04-25

**Authors:** Margarita Echeverri, David Anderson, Jacqueline M. Haas, Marc E. Johnson, Friar Sergio A. Serrano, Anna María Nápoles

**Affiliations:** 1 College of Pharmacy, Xavier University of Louisiana, New Orleans, LA 70125, USA; 2 Department of Mathematics, Xavier University of Louisiana, New Orleans, LA 70125, USA; danders2@xula.edu; 3 Multicultural Community Advisory Board, New Orleans, LA 70118, USA; jacquelineneworleans@gmail.com; 4 African American Cancer Community Advisory Board, Kenner, LA 70063, USA; mjohnson@fifthcircuit.org; 5 Latino Community Advisory Board, Hispanic Apostolate, Metairie, LA 70003, USA; saserrano@archdiocese-no.org; 6 Division of Intramural Research, National Institute on Minority Health and Health Disparities, National Institutes of Health, Bethesda, MD 20892, USA; anna.napoles@nih.gov

**Keywords:** health disparities, cancer screening, multidimensional framework, cancer literacy

## Abstract

The objective of this study was to evaluate the applicability of a multidimensional framework to explore factors associated with cancer literacy and its effects on receiving cancer screenings among diverse populations. Based on the conceptual framework, we developed and pilot-tested the Multidimensional Cancer Literacy Questionnaire (MCLQ) among 1500 individuals (African Americans, Latinos and Whites) in Louisiana. Exploratory factor analysis was used to identify the MCLQ underlying structure and predominant factors explaining each of the dimensions in the model. A total of 82 items (explaining 67% of the total variance) in the MCLQ were grouped into 20 factors associated with three key dimensions related to cancer literacy. Preliminary validity of the MCLQ was supported: Cronbach alpha for the scale score was 0.89 and internal consistency reliability coefficients for each factor were all above 0.67. The Facilitators Domain included five factors (28 items) that may positively influence individuals to have early-detection cancer screenings. The Barriers Domain included seven factors (26 items) explaining aspects that may negatively influence individuals to have cancer screenings. The Cultural Domain included eight factors (28 items) related to aspects that influence positively or negatively individuals’ perceptions regarding cancer as a disease, screenings and treatments. A multidimensional framework to study cancer literacy, including cultural attitudes, beliefs and practices, as well as facilitators and barriers, among diverse populations, will increase understanding of factors influencing individuals’ approach to cancer prevention and screening. Results will inform further testing of the multidimensional framework and questionnaire.

## 1. Introduction

Although research linking health literacy and health disparities is emerging [1,2], there is consensus that low health literacy leads to poorer health outcomes [3]. Health literacy is often included as a social determinant of health because of the interrelationships between education level, health literacy and health outcomes [4,5,6]. Specifically, as with low educational levels, low health literacy is associated with poorer health status, lower treatment compliance, increased emergency rooms visits and decreased ability to understand instructions and participate in decision-making [7,8]. Intervention studies that target low health literacy have sought to decrease health disparities on a number of varied behavioral outcomes, such as increasing healthcare service access or utilization, improving patient self-management skills or implementing disease-specific self-management plans. Other health disparities interventions that target health literacy have focused on increasing knowledge, self-efficacy, health communication and quality of life as well as reducing healthcare costs [2,9].

According to the National Academy of Sciences Roundtable on Health Literacy “it is the responsibility of health professionals to make sure all their patients (including those with low health literacy) truly comprehend the information they are being given [10].” Accordingly, one of the objectives of the 2020 Healthy People Initiative is to improve the health literacy of the population as a means to eliminate healthcare disparities (Objective HC/HIT-1) [11]. This objective is included as a key issue in the health and healthcare domain [7] and it is measured through three indicators that focus on increasing the number of people who report that their healthcare providers always (1) gave them easy-to-understand instructions, (2) asked them to explain the instructions given and (3) helped them in filling out forms. However, addressing low health literacy requires a more holistic approach that includes the complex interactions between patients, healthcare providers and healthcare systems.

It has been well documented that poor health literacy is an important barrier to cancer screening adherence [12,13,14,15]. Although studies have identified the impact of social determinants of health, such as cultural beliefs, health literacy and language on disparities in cancer screening rates among minority populations [16,17,18], there is substantial evidence that health literacy is a complex, multidimensional construct that cannot be measured using generic measures that do not address different domains related to management of specific chronic diseases such as cancer [19,20,21,22,23,24].

Several authors have conducted systematic reviews [25] on health literacy and cancer screening and contributed to the development of conceptual models that identify various domains of health literacy. Zarcadoolas and colleagues developed a multidimensional definition and model of health literacy that integrates and acknowledges the important roles of four types of literacy: fundamental literacy (ability to read, write, speak and work with numbers); science literacy (knowledge and abilities to understand scientific concepts including the rapid change of technology and uncertainty of science results); civic literacy (awareness of public issues, and skills needed to evaluate different positions and make decisions); and cultural literacy (abilities to recognize and understand social identity and collective beliefs and customs). The model is recommended to analyze health communication, improve interventions in health-related communications and develop assessment tools that allow the creation of profiles of people’s health literacy [26]. Sørensen and colleagues conducted a systematic review of definitions and conceptual frameworks of health literacy and identified four competencies related to health literacy that are required to navigate the healthcare system: Access (ability to seek, find and obtain health information); understand (ability to comprehend the health information that is accessed); appraise (ability to interpret, select and evaluate the health information that has been accessed); and apply (ability to use the information to make a decision) [27]. They applied the four competencies to build a 12-dimensional model of the impact of health literacy across three domains of the healthcare services continuum (promotion, prevention and treatment). Sørensen’s model distinguishes between distal (demographic situation, culture, language, political forces, etc.) and proximal (personal characteristics, social support, personal influences, media use, etc.) factors.

In a systematic review, Berkman and colleagues developed an analytic framework that delineates the relationships between health literacy skills, interventions and outcomes [2]. Building on the Integrative Theory, they describe a core set of variables (e.g., attitudes, health status, social norms, patient-provider relationships and self-efficacy) that explain individual’s behavioral intention (e.g., taking medication, changing lifestyle or having screening tests), and that combined with the adequate skills (e.g., knowledge, cognitive abilities, information seeking and decision-making) and removal of barriers (e.g., access to health insurance and language services,), predicts behavior change. Although they found mixed evidence supporting their model in studies of health literacy and cancer screenings, they concluded that there was moderate evidence that lower literacy is associated with decreased utilization of Pap smear screening for cervical cancer and mammography for breast cancer, and weak evidence for colon cancer screening. In support of the model, a systematic review of attitudes toward prostate cancer among African American men found that individual (knowledge, patient-provider communication, perception of personal risk and personal/family history of cancer), cultural (threat to masculine identity, fear of cancer, mistrust of the healthcare system and religious fatalism) and social (access to preventive care, income and education) factors influenced their decision to have prostate cancer screening [28].

Although these models are addressing multi dimensions related to health literacy, Zarcadoolas and Sørensen models do not focus on cancer literacy; Berkman’s analytic framework focuses only on interventions and outcomes instead of factors influencing cancer screening; and Pederson’s systematic review is narrowed to factors associated with prostate cancer screening among African American men. Building on these models and reviews, our approach is to go beyond people’s health literacy skills and traditional narrow conceptualizations and measurement of health literacy (reading, oral and numeracy) to investigate cancer health literacy specifically, and include other factors (motivation, self-efficacy, empowerment, socio-environmental influences) that might contribute to cancer screening disparities [29].

While no framework was found in the literature related to cancer literacy among diverse populations, in this study, we aimed to develop and test a new multidimensional framework of the effects of cancer health literacy on cancer prevention and screening behaviors among African Americans, English-speaking Latinos, Spanish-speaking Latinos and non-Latino whites that is comprehensive and includes cultural attitudes, beliefs and practices, as well as language and health literacy factors. The objective of this study is to identify the underlying structure and subscales of the Multidimensional Cancer Literacy Questionnaire (MCLQ) and test the preliminary validity of the multidimensional framework. The MCLQ focuses on predisposing factors that influence potential cancer screening mediators and outcomes. In this analysis, we focused on testing the structure and subscales of the predisposing factors only. Our rationale for focusing on this first and major portion of the framework is to apply data reduction techniques before linking these predisposing factors to potential cancer screening mediators and outcomes.

## 2. Materials and Methods

Based on the literature, in this study, we first, developed and described our conceptual framework, the Multidimensional Cancer Literacy Framework. Then, based on the Framework, we developed the Multidimensional Cancer Literacy Questionnaire (MCLQ). Then, we conducted a field test of the MCLQ through a self-administrated cross-sectional survey of diverse populations residing in New Orleans. Finally, we used data collected from the field test on factors associated with multiple domains related to cancer health literacy to examine the preliminary validity and internal consistency reliability of the measures and refine the Multidimensional Cancer Literacy Framework.

### 2.1. Development of Multidimensional Cancer Literacy Framework (MCLF)

The development of our conceptual framework, the Multidimensional Cancer Literacy Framework was informed by prior work, the Health Belief Model [30] and Dr. Zarcadoolas’ definition of health literacy [26]. The Health Belief Model (HBM), an individual-level framework, offers several constructs related to perceived barriers, benefits and risks that are useful and relevant for predicting cancer screening behaviors. According to the HBM model, the likelihood that individuals engage in cancer screening behaviors is influenced by their beliefs about cancer and screening risks (perceived susceptibility) and the severity of the disease and the possible harm and benefits of the screening tests (perceived seriousness).

**Figure 1:**
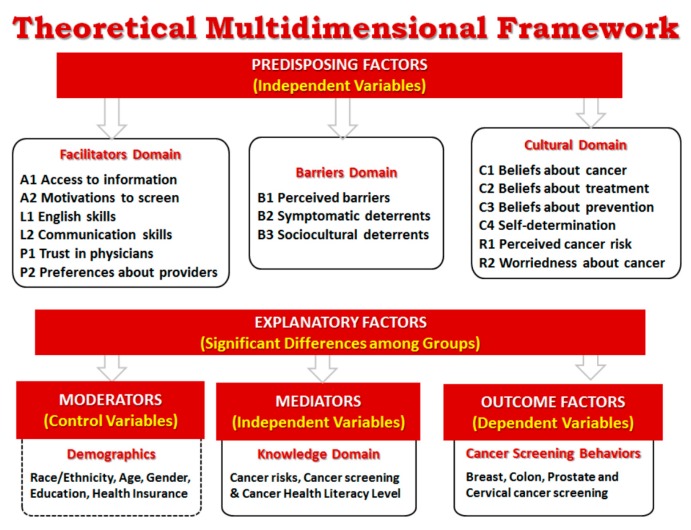
Multidimensional conceptual framework.

In this study, we also apply Dr. Zarcadoolas’ broad definition of health literacy as “the wide range of skills and competencies that people develop over their lifetimes to seek out, comprehend, evaluate, and use health information and concepts to make informed choices, reduce health risks, and increase quality of life” [26] (p. 55). This definition was used to elucidate the specific skills, competencies and use of health information related to cancer literacy that could affect cancer screening rates. The multidimensional framework (Figure 1) expands on this prior work to include cultural factors and issues such as English language skills and trust in physicians that are especially relevant to minority communities.

Drawing from the literature review [31,32,33,34,35,36], items for the MCLQ were organized into three inter-related subdomains that fall under the domain of Predisposing Factors: The Facilitators Domain, the Barriers Domain and the Cultural Domain (Figure 1). The Facilitators Domain includes six factors related to enablers supporting or facilitating cancer screening such as motivation to screen, access to information, English and communication skills, and trust in physicians and preferences about healthcare providers. The Barriers Domain includes three factors related to barriers or obstacles to screening including perceived barriers and symptomatic and sociocultural deterrents [31]. The Cultural Domain includes six factors to measure participants’ beliefs about cancer as a disease, cancer treatment and cancer screening as a preventive measure; as well as participants’ beliefs influencing the way they make decisions about cancer (self-determination) and their perceptions about own risk and worriedness about having cancer.

The Outcomes Factors include items that measure participants’ screening behaviors for the more common cancers for both women and men. Additionally, the framework includes items to measure specific demographic characteristics that may influence decisions about cancer screening such as race/ethnicity, age, gender, education and type of health insurance (moderators). Factors in the Knowledge Domain include potential mediators of cancer screening outcomes including items to measure cancer literacy level and knowledge and understanding of cancer risks and early detection screening methods. These items act as mediators of the relationship between the predisposing factors and cancer screening behavior outcomes [2].

### 2.2. Development of Multidimensional Cancer Literacy Questionnaire (MCLQ)

A literature review of tools measuring cancer health literacy and related constructs resulted in the identification of main domains related to cancer health literacy (Table 1). Selected subscales and/or individual questions from these tools were adapted, as needed. Questions were organized into the preliminary domains that make up our theoretical multidimensional framework (Figure 1) and focus on participants’ knowledge and perceptions about: cancer (causes, types, risks and symptoms); prevention (screenings and healthy behaviors); and treatments (options and access); as well as their cancer screening behaviors. Special attention was given to the operational definition of variables and constructs, the types of questions included in the questionnaire and response options. An Expert Panel was created to revise the MCLQ and determine the applicability of scales, subscales or individual items drawn from the literature to diverse populations.

The MCLQ was developed in Spanish and English and pilot-tested using cognitive interviews with 20 volunteers from each of the Latino, African American and White communities in Louisiana. Participants were recruited by members of the Community Advisory Boards (CABs) who have had a long-term relationship with the Principal Investigator (PI) in each targeted community. These interviews were conducted by the PI during community meetings specifically scheduled for this purpose. During the cognitive interviews, participants self-administered the MCLQ, and then were asked to provide feedback about the questionnaire (objective, consistency, clarity, length, applicability, etc.), the wording, response options and organization of the questions and the appropriateness of the incentive offered ($30). The MCLQ was revised to maximize comprehension based on the results of the cognitive interviews. The revised tool was reviewed again by the Expert Panel for final approval prior to the field test.

### 2.3. Participants and Data Collection Procedure for Field Test

This project employed community based participatory research (CBPR) methods and was approved by the Xavier University of Louisiana’s Institutional Review Board (IRB). Inclusion criteria for the field test were: ages 25 years old or older and living in Louisiana. Recruitment was stratified to obtain similar numbers by race (Latinos, African American and Whites) and gender (male/female). Efforts were made to include participants of varying educational levels (primary school or less; some high school, high school degree; some college studies; or a bachelor’s or more advanced diploma).

Flyers inviting participants to the study and explaining the purpose of the study, dates and locations of data collection events, time commitment, incentives and contact information were delivered by CAB members and other community leaders to businesses, organizations and centers serving the different communities.

At the meetings, the Principal Investigator (PI) explained the rationale and objectives of the study and used a script to obtain verbal consent of participants to complete the one-time survey. Participants meeting the inclusion criteria and willing to participate in the study were enrolled in the study. A copy of the script was given to each participant to keep. In order to maintain anonymity, questionnaires were numbered with a consecutive number and did not include any personal identifiers. Questionnaires were hand delivered by the PI and community outreach leaders, who were attentive to answer any questions participants had as they self-administered the questionnaire. Completion of the questionnaire took on average 30 min. Survey responses were entered into an Excel file by community members hired as part of the study and the Community Outreach Coordinators reviewed data entry against original questionnaires to check for accuracy.

### 2.4. Statistical Analysis to Refine the MCLQ

Factor analysis is one of the most commonly used techniques of data reduction in social science where a large set of observed variables is reduced to a smaller set of hypothetical or latent variables (factors) [37]. There are two different methods for factor analysis. EFA is a tool intended to help generate a new theory (theory-building) and estimate the unknown structure of the data when there is no prior theory about the factor structure of the data. On the other hand, confirmatory factor analysis (CFA) is used to test an existing theory (theory-testing), that is, to examine if an a priori model of the underlying structure of the constructs (constrained and unconstrained) fits the new data adequately [37,38].

Considering that the MCLQ is a new tool, exploratory factor analysis (EFA) with principal components and Varimax rotation was used as a data reduction tool to identify the underlying structure and subscales of the MCLQ and discover predominant factors (constructs) explaining each of the domains included in the multidimensional framework (Figure 1). Recommended sample size when conducting principal component analysis depends on the number of items in the questionnaire (participant-to-items ratio). Many authors recommend a 10:1 ratio as the rule of thumb [39]. The original MCLQ included 99 items (Table 1), thus, the targeted sample size for this study was designed to yield an adequate participant-to-items ratio suitable for application of factor analysis techniques to explore the underlying structure of the MCLQ. We used the following criteria to determine the validity of the resulting constructs (scales): (1) total variance explained; (2) eigenvalue > 1; (3) eliminating items with low structure coefficient (loads < 0.5); (4) eliminating items that showed high cross loading (>0.4), that is, loaded significantly on more than 1 factor (discriminant validity); and (5) eliminating items with low extraction communality (h2 < 0.40) [38]. Additionally, Cronbach alpha coefficients were used to assess the internal consistency reliability of the scales. During the reliability analysis, the following requirements also were verified: (1) whether the item-total correlation corrected for overlap (item discrimination) in new developed scales was > 0.30 [40]; (2) whether the elimination of an item caused the alpha to increase; (3) whether a reduced range of responses was observed in an item; and, (4) whether item means were extreme. SPSS, version 13.0.1 (SPSSInc, Chicago, IL) and R were used to carry out the data analyses.

Additionally, considering that we have a large sample (*n* = 1500), and as a way to test the preliminary validity of the results obtained, we conducted a secondary analysis running EFA on the first half of the data (750 randomly selected individuals) and CFA, using Diagonally Weighted Least Squares (DWLS), on the second half of the data [41].

## 3. Results

The sample consisted of 1500 adults between the ages of 25–94 (Mean = 48.3 years), equally distributed by race/ethnicity (500 African Americans, 500 Latinos and 500 Whites), and gender (50% male and 50% women). Educational level was roughly distributed with a lower percentage of participants having some high school or lower (20%) and most participants having a high school diploma (29%), some college (31%) or advanced degrees (20%). Greater details on the demographic characteristics of the sample have been previously published [42] and are included (Table 2) with permission of the authors.

### 3.1. Multidimensional Framework Exploration

**Figure 2:**
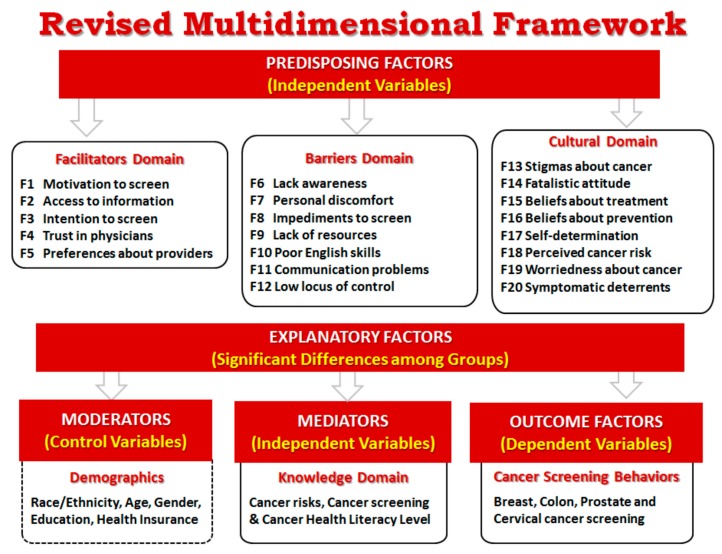
Revised multidimensional framework.

The exploratory factor analysis resulted in 20 factors (Figure 2) instead of the 15 initially considered in the conceptual framework (Figure 1). A total of 82 out of the 99 items in the MCLQ explained 67% of the total variance (Table 3). Based on the criteria specified in the methods section, 17 items were eliminated during the factor extraction process: six of them because they were not applicable to the entire sample population (male, female or Spanish speakers) and the other 11 because they did not meet the methodological criteria. Cronbach alpha for the total scale score was 0.89, and above 0.67 for all the subscales except for three (F12-*Low Locus of Control*, F15-*Beliefs about Treatment* and F17-*Self-determination*). Results of the Bartlett test statistic for sphericity and the Kaiser–Meyer–Olkin (KMO) measure of sampling adequacy (chi-square = 47930.2; *p* < 0.0001 and KMO value = 0.877) confirmed that factor analysis is suitable to be used as the statistical technique in this study.

Following the conceptual framework, the resulting factors were organized into three main domains or scales (Table 3, Figure 2). The five additional factors found during the exploratory analysis consisted of groups of items that separated as independent factors. In the Barriers Domain, the factor B1-*Perceived Barriers* separated into two factors F6-*Lack of Awareness* and F7-*Personal Discomfort* and the factor B3-*Sociocultural Deterrents* separated into two factors F8-*Impediments to Screen* and F9-*Lack of Resources*. In the Cultural Domain, three factors separated each into two different groups. C1-*Beliefs about Cancer* separated into F13-*Stigmas about Cancer* and F14-*Fatalistic Attitude*; C3-*Beliefs about Prevention* separated into F3-*Intention to Screen* (which was moved to the Facilitators Domain) and F16-*Beliefs about Prevention*; and C4-*Self-determination* separated into F12-*Low Locus of Control* (which was moved to the Barriers Domain) and F17-*Self-determination*.

### 3.2. Facilitators Domain

Five factors consisting of 28 items explaining 21% of the total variance were classified in the Facilitators Domain (Table 4). This Domain considers aspects that may influence positively individuals to have early detection (preventive) cancer screenings. F1*-Motivation to Screen* includes seven items explaining reasons to get screening such as getting a reminder, having a symptom, having company (social support), getting it for free, etc. During the analysis, two items in this factor were deleted: The item a-“I asked the doctor to order the test” was deleted because of low factor loading, and the item b-“an interpreter was available during the exam” was deleted because it was cross loaded with F10. F2*-Access to Information* includes eight items related to the use of different mediums to get information about cancer screenings such as the radio/TV, Internet, newspapers, health fairs, friends, doctors, etc. F3*-Intention to Screen* includes five items reinforcing regular screening practices as an early detection mechanism that may save lives, that should be practiced by some people regularly, and that may be perceived as regular care. F4*-Trust in Physicians* includes four items that may influence patients’ trust such as the cultural and racial background of their healthcare providers and their perceptions about the capacity that doctors have to give advice and cure diseases. F5*-Preferences about Providers* includes four items focused on the main characteristics (race, gender, language and religion) that patients consider when looking for a healthcare provider.

As initially considered in our conceptual framework we expected to discover six factors in this domain (Figure 1) but in the revised model, five factors were identified (Figure 2, Table 4). While four of the original factors (F1-*Motivation to screen*, F2-*Access to information,* F4-*Trust in Physicians* and F5-*Preferences about Providers*) were supported during the analysis, the other two factors (L1-*English Skills* and L2-*Communication Skills*) were moved to the Barriers Domain and were renamed as F10-*Poor English Skills* and F11-*Communication Problems*. Additionally, a new factor, F3-*Intention to Screen,* was included in the Facilitators Domain. This F3 factor is the result of a group of items in the C3-*Beliefs about Prevention* that grouped independently and was originally included in the Cultural Domain (Figure 1, Table 1).

### 3.3. Barriers Domain

Seven factors consisting of 26 items explaining 22% of the total variance were classified in the Barriers Domain (Table 5). This Domain considers aspects that may discourage individuals from having early detection cancer screenings. F6*-Lack Awareness* includes two items that are important barriers to screening such as not having a doctor to order the test or not knowing if there is a need for the test. F7*-Personal Discomfort* includes five items related to individuals’ reluctance to be screened such as being afraid of the procedures or results, lack of trust in the tests and aversion of having one’s private parts touched. F8-*Impediments to Screen* includes six reasons why individuals experience difficulties in making an appointment for early cancer detection examinations such as forgetting and/or not receiving a reminder to make the appointment, not knowing when is time to have the screening or where to go, having difficulties scheduling the appointment, or having long wait times to get an appointment for a screening test. F9*-Lack of Resources* includes five items related to lack of support to get the screening such as lack of insurance, money and transportation or need of company (social support) or child care services. F10*-Poor English Skills* includes three items measuring individual English skills (speaking, writing and reading) that are important when navigating the healthcare system. F11*-Communication Problems* includes three items related to communication barriers during the medical encounters such as having a healthcare provider who speaks very fast or has a heavy accent as well as difficulties understanding medical forms such as consent and health history forms. F12*-Low Locus of Control* includes two items measuring patients’ uncertainties when making the screening decision because of lack of knowledge or being afraid to offend the doctor if not following the recommendations.

As initially considered in our conceptual framework we expected to include in this domain only three factors related to barriers (Figure 1), however, we found instead seven factors (Figure 2), including two new identified factors and three that were moved from the other Domains: F6-*Lack Awareness* and F7-*Personal Discomfort* were initially included together in the B1-*Perceived Barriers*; F8-*Impediments to Screen* and F9-*Lack of Resources* where initially included together in the *B3-Sociocultural Deterrents; F10-Poor English Skills* and *F11-Communication Problems* were originally considered in the Facilitators Domain as positive factors to cancer screening but were moved to the Barriers Domain and renamed to indicate their influence as barriers instead of facilitators; and F12-*Low Locus of Control*, which items were initially included as part of the C4-*Self-determination* factor in the Cultural Domain was classified as an independent factor in the Barriers Domain instead.

### 3.4. Cultural Domain

Eight factors grouping 28 items and explaining 24% of the total variance were classified in the Cultural Domain (Table 6). This Domain considers cultural aspects that may influence perceptions regarding cancer as a disease as well as cancer screenings and treatments. F13-*Stigmas about Cancer* and F15*-Beliefs about Treatment,* include two items each that relate to participants’ self-explanations about reasons to get cancer and likelihood that it can be cured. F14-*Fatalistic Attitude* includes three items related to perceptions that cancer is a death sentence. F16-*Beliefs about Prevention* includes five items explaining participants’ perceptions of no need for cancer prevention screening in general. F17-*Self-Determination* includes four items measuring participants’ approaches to making their own decisions and taking control of their health decisions. F18-*Perceived Risk* includes four items measuring participants’ perceived risk of having stomach, liver, colon, or skin cancer in their lifetime. F19-*Worriedness about Cancer* includes four items measuring participants’ fears of having stomach, liver, colon, or skin cancer in their lifetime. F20*-Symptomatic Deterrents* includes four items explaining participants’ reasons for not having cancer prevention screenings in particular.

As initially considered in our conceptual framework, we expected to include in this domain six factors (Figure 1) but instead we found eight factors (Figure 2) including one new factor and one that was moved from another Domain. C1-*Beliefs about Cancer* that had initially seven items (Table 1), separated into two factors F13-*Stigmas about Cancer* (2 items) and F14-*Fatalistic Attitude* (3 items). The other two items (“It seems like almost everything causes cancer” and “Everybody has cancer but only some develop the disease”) were deleted because they had low factor loadings. C2-*Beliefs about Treatment* had five items (Table 1) but three of them were deleted (”All you need to beat cancer is a positive attitude, no treatment”, “There is a cure for cancer but it is only available to the rich and privileged” and “There is nothing to do if diagnosed with cancer”) because of low and cross loadings with other factors, so it was renamed as F15-*Beliefs about Treatment*. C3-*Beliefs about Prevention* had initially ten items (Table 1) and they divided into two factors F3-*Intention to Screen* (5 items) and F16-*Beliefs about Prevention* (5 items). F3 was moved to the Facilitators Domain because of its positive impact in screening. C4-*Self-determination* had eight items (Table 1) that separated into two factors F12-Low locus of control (2 items) that was moved to the Barriers Domain, and F17-*Self-determination* (4 items). The other two items (“If have a symptom, would go to the doctor to get it checked immediately” and “I feel that some doctors treat me differently because of my different racial background”) were deleted because of low factor loadings. The other original factors in the Cultural Domain (R1-*Perceived Cancer Risk* and R2-*Worridness about Cancer*), each had seven items (Table 1) and were renamed as F18 and F19. Three items related to breast, cervical and prostate cancer were deleted in each of these two factors because they were not applicable to the entire population (only male or female participants). Additionally, F20-*Symptomatic Deterrents* was moved from the Barriers Domain because the items refer more to beliefs than barriers.

### 3.5. Secondary Analysis of Preliminary Validity of the Constructs

As explained in the methodology, in order to explore better our framework, we also conducted exploratory factor analysis (EFA) and confirmatory factor analysis (CFA) by randomizing half of the sample (*n* = 750) to each analytic approach. The CFA confirmed the preliminary validity of 12 of 20 factors initially found in the full EFA (Figure 2). Items in the F1-*Motivation to screen* (9 variables) were not included in these secondary analyses because these questions were asked only of participants who have had any kind of cancer screening (branched questions). Specifically, the CFA did not confirm four factors that had only two items (F6-*Lack of Awareness*, F12-*Low Focus of Control*, F13-*Stigmas about cancer* and F15-*Beliefs about treatment*), one factor (F17-*Self-determination*) for low Cronbach’s Alpha and one factor (F11-*Communication problems*) because of low h^2^ scores. In addition, all of these factors that were not confirmed had relatively low explanatory power. Interesting, the CFA grouped together the items in F8-*Impediments to screen* and F9-*Lack of resources* meaning that both factors may complement each other.

The CFA showed that the model including only the twelve confirmed factors (F2, F3, F4, F5, F7, F8–9, F10, F14, F16, F18, F19, F20) had excellent overall validity: Chi-square test = 6922.24 (degree of freedom = 2473); *p* < 0.001; Comparative Fit Index (CFI) = 0.970; Tucker Lewis Index (TLI) = 0.967; Root Mean Square Error of Approximation (RMSEA) = 0.030 (90% CI: 0.028–0.032); and Standardized Root Mean Square Residual (SEMR) = 0.049. The total scale explained 50% of the variance and had a Cronbach alpha of 0.841 (95% CI: 0.819–0.859). All of the factors except F9-*Fatalistic Attitude* had a Cronbach alpha above 0.8.

## 4. Discussion

The objective of this study was to field test the MCLQ and identify the underlying structure of the data, and whether this structure is consistent with our conceptual framework for diverse populations (Figure 1). Results confirmed three main domains (Facilitators, Barriers and Culture) and the informed revision of the framework (Figure 2, Table 3). Key findings are discussed next.

The F3-*Intention to Screen* construct was initially considered to be a part of the cultural domain because the items referred to beliefs regarding cancer prevention and screening that were thought to be culturally-based (Figure 1). Upon closer inspection, the items included in this factor (Table 4) are more related to subjective norms that function as “facilitators” for the adoption of cancer screening: “Routine cancer screening shows that people care for their health and their families”; “All people should have regular cancer screenings”; “Cancer screenings help to save lives”; and “Friends of my age have cancer screenings regularly.” These findings are consistent with those from another study that found that perceived subjective norms (“it is important for me to comply with what my close friend believes”, “it is important for me to do what my parents think is appropriate” and “the important people in my life believe colon screening can help prevent colon cancer”) predicted colon cancer screening [43].

In our theoretical framework (Figure 1), we expected that items in F6-*Lack Awareness* and F7-*Personal Discomfort* would group together considering that they all were related to personal issues that would be barriers to regular cancer screening. However, instead, they grouped into two different factors. Items in F6 focus on issues that are not under the control of the person while items in F7 focus more on personal concerns that are managed at the individual level. Similarly, we expected that items in F8-*Impediments to Screen* and F9-*Lack of Resources* would also group together on the same factor, considering that they relate to real barriers that participants have had to regular screening. However, items related to barriers to get the screening appointment grouped with F8 while lack of resources separated into an independent factor, F9. Interestingly, in the secondary analysis conducted using CFA, these two factors grouped together as initially expected.

Additionally, we expected that items related to English skills (F10) were not going to stay together as a strong factor. Our assumption was that these items were important only for Latino immigrant participants who may have poor English skills and needed interpreter services. However, during the analysis items related to the need (barrier) and use (facilitator) of interpreters were deleted because they were cross loaded with this factor. Poor English skills may not be only a concern for Latino immigrants. A report about adult literacy in the U.S. found that “U.S.-born adults make up two-thirds of adults with low levels of English literacy skills” [44] [p. 2], and the American College Testing (ACT), one of the major admission tests for college in U.S., found that only 59% of the high school class of 2019 reached the minimum college readiness benchmark in English [45].

Initially, we included items in F12-*Low Locus of Control* as cultural aspects influencing individual’s capacity to make decisions (C4-Self-determination, Table 1). However, considering that they separated themselves into an independent factor and when looking at the statements (“I would offend my doctor if I were to make my own decisions about my health”, and “I don’t know enough to make my own medical decisions”), it makes sense that they are included in the barriers domain instead (Table 5).

During the literature review, it was not clear about the different impact that perceived cancer risk (F18) and worriedness about cancer (F19) would have on cancer screening (Table 6). While some authors choose one construct over the other, we decided to keep both in order to be able to select the one that best matches the model. Interestingly, both concepts grouped as independent and strong factors. These results support that patients’ perceptions about risk are different than cancer worry. However, items related to risk and worry of men-related cancer (prostate) and women-related cancers (breast and cervical) had to be deleted during the analysis because they were not applicable to the entire sample. According to Klein and Stefanek [46], individual decisions such as avoiding known risk factors, utilizing screening tests, undergoing cancer testing and making treatment decisions are “tied inextricably to comprehension and perceptions of personal risk.” (p. 147) Specifically, they argue that people’s perception of risk or worriedness of getting the disease is influenced by: “the frequency (e.g., how many people smoke); the covariation (e.g., how many smokers get lung cancer); the similarity (e.g., how many of my smoking friends have gotten lung cancer); and normativeness (e.g., how unusual is a smoker to die from something else than lung cancer)” [p. 151]. Interestingly, when looking at the perception of risk, people usually estimate their risk to be lower than average. However, when looking at the worriedness about having the disease, Klein and colleague explain that people usually focus more on the 8 people who died because of the disease than on the 92 who did not, and their personal behaviors (e.g., If I smoke, how much I smoke, what kind of cigarettes I smoke, etc.) [46]. In summary, worriedness is a function of both how people view their own risk and how they compare their risk with that of others [47].

When conducting factor analysis, it is recommended that factors with two items be interpreted with caution, especially when the variables have a low correlation with each other (r < 0.70) [38,39]. In our case, four factors (F6-*Lack Awareness*, F12-*Low Locus of Control*, F13-*Stigmas about Cancer* and F15-*Beliefs about Treatment*) had only 2 items each (Table 5 and Table 6). Although these items contribute little to the total variance explained by the model (Table 3), all of them, except F12, have factor loadings of over 0.5, meeting criteria for inclusion in the revised framework. Although these factors were not confirmed in the CFA analysis, considering that our objective is to examine the preliminary validity of the conceptual framework and the importance of these factors in individuals screening behaviors, instead of deleting the items in these four factors, we decided to keep them for further testing.

Important limitations of this study need to be considered. As this study is a preliminary exploration of a new developed conceptual framework and its respective questionnaire, results need to be interpreted with caution. In addition, three of the subscales, F12-*Low Locus of Control*, F15-*Beliefs about Treatment* and F17-S*elf-Determination* demonstrated marginal internal consistency reliability (Cronbach alphas). Based on the conceptual importance of these constructs with respect to cancer screening, especially among diverse populations, we recommend that researchers continue to develop and test the conceptual and psychometric adequacy of these measures. Although the tool is lengthy (82 items), the subscales could be used independently; however, caution is recommended when using those scales for which validity was not confirmed in the secondary analysis of the data. We suggest that researchers continue to refine and test the subscales and items of the MCLQ to improve their psychometric properties. This future work could include known-groups validity testing to help determine if MCLQ scores discriminate among subgroups of diverse populations known to differ on some of these constructs.

## 5. Conclusions

In summary, we identified 20 factors related to cancer health literacy that act as facilitators (positive) or barriers (negative) or that address cultural differences to engagement in cancer screening for early detection. Results provide preliminary evidence supporting the use of a multidimensional framework in the development of new tools for advancing research in cancer health literacy among diverse populations. A multidimensional framework to study cancer health literacy, including cultural attitudes, beliefs and practices, as well as facilitators and barriers, will increase understanding of factors influencing individuals’ approaches to cancer prevention and screening. Results will inform further testing of the multidimensional framework and questionnaire and their utility for addressing cancer screening disparities.

## Figures and Tables

**Table 1:** Theoretical Multidimensional Cancer Literacy Framework (MCLF): Domains, factors and items.

Domains and Factors	Item Content	Sources of Items or Item Themes
**Facilitators Domain**		
E1. Motivations to screen (9 items) ^1^	I have had regular cancer screening because a-I asked my doctor to order the test; b-An interpreter was available during the exam; c-The doctor/nurse recommended it; d-A family member or friend recommended it; e-I got a reminder card in the mail; f-I had a symptom that made me worry; g-I did not have to pay for it; h-I had the money to pay for the exam; i-I had somebody to go with me to the appointment	a–h Adapted from Buki [32]; i-added by participants in the pilot-test
E2. Access to information (8 items) ^2^	How much information about cancer have you received from a-Doctors or other health professionals; b-Family or friends; c-Newspapers or magazines; d-The radio or television; e-The Internet; f-Religious organizations and leaders; g-Government health agencies; h-Community health fairs	Adapted from HINTS [34]; Buki [32]
L1. English skills (3 items) ^3^	How well do you a-speak English; b-read English; c-write English	Adapted from HINTS [34]
L2. Communication skills (3 items) ^1^	a-It is difficult for me to fill out medical forms; b-I have a hard time understanding when health professionals speak to me quickly; c-I have had difficulties understanding doctors who come from other regions and have a different English accent	a-Adapted from Buki [32]; b-Adapted Morris [36]; c-Recommended by Latino participants in pilot-test
P1. Trust in Physicians (4 items) ^4^	a-How much do you trust your doctor’s decisions about which medical screenings are best for you; b-How much do you trust doctors of a different race/ethnicity from you; c-How much do you trust doctors of same cultural background; d-How effective are doctors at curing illness	all-Adapted from Mayfield [35]; Buki [32]
P2. Preferences about Providers (4 items) ^1^	a-I prefer my doctor to be of my same gender; b-I prefer my doctor to be of my same racial/ethnic and cultural background; c-I prefer a doctor who speaks my language; d-I prefer my doctor to be of my same religion	Added by participants in pilot-test and adapted by authors’ experience with previous research
**Barriers Domain**		
B1. Perceived barriers to screening (7 items) ^1^	The following would be barriers or obstacles for me to have regular cancer screenings a-The doctor does not order the tests; b-Being unsure if there is a need for testing; c-Lack of trust on these cancer tests; d-Being afraid the test will cause harm if it is not well done; e-Being afraid the test will cause cancer; f-Dislike having exams in those parts of the body; g-Do not want to know if cancer is present	a–g Adapted from Buki [32]
B2. Symptomatic deterrents (4 items) ^1^	There is no need to have cancer screenings when a-Feeling healthy; b-Having several normal screening test results; c-Not feeling anything abnormal (symptoms); d-Being too young or too old	a–c Adapted from Betancourt [31]; d-added by participants in the pilot-test
B3. Sociocultural deterrents (13 items) ^5^	I have had the following problems to get the cancer screening exams a-Do not know where to go for the exam; b-Have forgotten to make the appointment; c-Have had problems making the appointment; d-Do not know if it is time to have the exams; e-Have not received a reminder postcard; f-Waiting too long for an appointment; g-Not able to get time off work; h-Lack of transportation to go to the appointment; i-Need somebody to take care of my children or family; j-Lack of health insurance; k-Lack of money to pay for the exam; l-Need an interpreter during the appointment; m-Need a friend/family to go with me to the appointment	a,c,e,g–k Adapted from Betancourt [31]; b,d,f, Adapted Buki [32]; l–m Added by Latino participants
**Cultural Domain**		
C1. Beliefs about cancer as a disease (7 items) ^1^	a-It seems like almost everything causes cancer; b-Cancer is due to bad luck; c-Cancer is a punishment from God; d-Cancer is the worst thing that can happen to a person; e-Cancer is a deadly disease; f-When I think of cancer, I automatically think of death; g-Everybody has cancer but only some develop the disease	a-Adapted from Morris [36]; HINTS [34]; b-Adapted Buki [32]; c-Adapted Buki [32]; d-e Adapted Betancourt [31]; f-HINTS [34]; g-added by participants in pilot-test
C2. Beliefs about cancer treatment (5 items) ^1^	a-All you need to beat cancer is a positive attitude, no treatment; b-Treating cancer with surgery can cause it to spread throughout the body; c-Cancer can be only cured if it is God’s will; d-There is a cure for cancer but it is only available to the rich and privileged; e-There is nothing I can do to change my fate if I find out I have cancer.	a-b Adapted from Gansler [33]; c-Added by participants in pilot-test; d-Recommended by participants in pilot-test; e-Adapted HINTS [34]
C3. Beliefs about screening as a preventive measure (10 items) ^1^	a-It is not necessary to have cancer screening regularly because it is in God’s hands anyway; b-It is not necessary to have cancer screening regularly because everyone will eventually die of something anyway; c-It is not necessary to have cancer screening regularly because if you are meant to get cancer you will get it no matter what you do; d-There are so many recommendations about preventing cancer; it’s hard to know which ones to follow; e-There is not much people can do to lower their chances of getting cancer; f-Cancer screenings help to save lives; g-Other people my age have cancer screenings regularly; h-All people should have regular cancer screenings; i-Routine cancer screening is a way to show that people care for their health and their families; j-It is possible for me to get cancer during my lifetime	a–b Adapted from Betancourt [31]; Morris [36], HINTS [34]; Buki [32]
C4. Self-Determination (8 items) ^1^	a-I feel comfortable checking my own body for signs of health problems; b-If I noticed a symptom, I would go to the doctor to get it checked immediately; c-If I found out I have cancer, I would seek a second opinion about my condition and treatment options; d-I would offend my doctor if I were to make my own decision(s) about my health; e-I don’t know enough to make my own medical decisions; f-I’d rather be given many choices about what’s best for my health than have the doctor make the decision for me; g-Sometimes, there are good reasons not to follow the advice of a doctor; h-I feel that some doctors treat me differently because of my different racial background	a–h Adapted from Buki [32]
R1. Perceived cancer risk (7 items) ^6^	Compared to other people my age and my same gender, I think that my risk of getting the following cancers are a-Breast cancer; b-Cervical cancer; c-Colon cancer; d-Stomach cancer; e-Liver cancer; f-Skin cancer; g-Prostate cancer	Adapted from HINTS [34]
R2. Worriedness about cancer (7 items) ^7^	How often do you worry about getting one of the following cancer types in your lifetime a-Breast cancer; b-Cervical cancer; c-Colon cancer; d-Stomach cancer; e-Liver cancer; f-Skin cancer; g-Prostate cancer	Adapted from HINTS [34]

^1^ Items beginning with the following phrase: ‘‘How much do you agree or disagree with the following statements.” Response choices were: strongly disagree; slightly disagree; undecided; slightly agree; strongly agree. ^2^ Response choices were: none, a little, a fair amount, enough, a lot. ^3^ Response choices were: not at all; a little well; somewhat well; well; very well. ^4^ Response choices were: not at all; a little bit; some; quite a bit; a lot. ^5^ Response choices were: never; rarely; sometimes; frequently; always. ^6^ Response choices were: much lower, lower, about the same; higher; much higher; no applicable. ^7^ Response choices were: never; few times; fairly often; many times; always, no applicable.

**Table 2:** Demographics of Participants ^1^.

	African Americans ^2^		Latinos ^3^		Whites ^4^		Total	
	*n*	%	*n*	%	*n*	%	*N*	%
**Total Participants**	500	33.3	500	33.3	500	33.33	1500	100.0
**Gender**								
Male	250	50.0	250	50.0	250	50.0	750	50.0
Female	250	50.0	250	50.0	250	50.0	750	50.0
**Age**								
25–40	135	27.0	211	42.2	181	36.2	527	35.1
41–55	169	33.8	150	30.0	143	28.6	462	30.8
56+	196	39.2	139	27.8	176	35.2	511	34.1
**Education**								
Primary school or lower	11	2.2	112	22.4	3	0.6	126	8.4
Some High School	76	15.2	74	14.8	29	5.8	179	11.9
High School Diploma	187	37.4	111	22.2	132	26.4	430	28.7
Some college or vocational diploma	152	30.4	133	26.6	175	35.0	460	30.7
Bachelor or advanced degree	74	14.8	70	14.0	161	32.2	305	20.3

^1^ Table copied with permission from the authors [42]. ^2^ Non-Hispanic Blacks. ^3^ Hispanics of any race. ^4^ Non-Hispanic Whites.

**Table 3:** Revised Multidimensional Cancer Literacy Framework (MCLF): Domains and factors.

Domains and Factors	% of Variance	N	Mean ^1^	SD	Cronbach’s Alpha	Number of Items
**Facilitators Domain**	21.04					28
F1 Motivation to screen ^2^	4.237	1104	2.71	1.094	0.813	7
F2 Access to information	6.126	1500	2.49	1.064	0.910	8
F3 Intention to screen	3.956	1500	3.92	0.995	0.821	5
F4 Trust in physicians	3.494	1500	3.87	0.837	0.845	4
F5 Preferences about providers	3.230	1500	2.54	1.154	0.822	4
**Barriers Domain**	22.41					26
F6 Lack awareness	1.970	1500	2.89	1.378	0.757	2
F7 Personal discomfort	3.961	1500	2.20	1.096	0.833	5
F8 Impediments to screen	4.881	1500	1.97	1.047	.866	6
F9 Lack of resources	3.530	1500	1.78	1.022	0.836	5
F10 Poor English skills	4.209	1500	3.83	1.527	0.978	3
F11 Communication problems	2.127	1500	2.46	1.214	0.670	3
F12 Low locus of control	1.731	1500	2.92	1.256	0.480	2
**Cultural Domain**	23.49					28
F13 Stigmas about cancer	1.999	1500	1.48	0.918	0.755	2
F14 Fatalistic attitude	2.586	1500	3.16	1.259	0.750	3
F15 Beliefs about treatment	1.829	1500	2.70	1.262	0.517	2
F16 Beliefs about prevention	3.561	1500	1.98	0.934	0.776	5
F17 Self-determination	2.225	1500	3.56	0.914	0.569	4
F18 Perceived cancer risk	3.870	1500	2.77	0.868	0.900	4
F19 Worriedness about cancer	3.976	1500	1.96	1.086	0.922	4
F20 Symptomatic deterrents	3.448	1500	2.52	1.292	0.856	4
**Total Scale**	66.95	1104			0.888	82

^1^ Range 1–5. ^2^ Data from 1104 participants who have had any kind of cancer screening.

**Table 4:** Factors and items in the Facilitators Domain.

Factors and Items	Item Description	Factor Load	Mean ^1^	SD	CITC	h^2^
**F1 Motivation to screen (7 items)**						
F1e_GotRemainder	Received a reminder (mail/call)	0.730	2.69	1.386	0.620	0.579
F1g_WasFree	Free service	0.697	2.64	1.377	0.574	0.524
F1f_HadSymptom	Had a symptom	0.691	2.70	1.392	0.574	0.530
F1i_GotCompany	Had company to go to the appointment	0.671	2.45	1.347	0.550	0.544
F1c_DoctorOrdered	The doctor/nurse recommended it	0.659	3.33	1.392	0.520	0.528
F1d_FriendRecommended	A family/friend recommended it	0.657	2.55	1.309	0.545	0.497
F1h_HadMoney	Had the money to pay for the exam/copayment	0.600	2.64	1.359	0.471	0.412
**F2 Access to information (8 items)**						
F2d_InfoFromRadioTV	From the radio or television	0.819	2.71	1.292	0.756	0.699
F2g_InfoAgenciasGno	From government health agencies	0.814	2.20	1.350	0.750	0.697
F2c_InfoFromNewspaper	From newspapers, bulletins, magazines	0.811	2.62	1.311	0.745	0.706
F2h_InfoFromHealthFairs	From community health fairs	0.796	2.27	1.392	0.716	0.693
F2f_InfoFromNGOs	From religious and community organizations	0.783	2.06	1.345	0.700	0.728
F2e_InfoFromInternet	From the Internet	0.769	2.70	1.413	0.691	0.668
F2b_InfoFromFriends	From family or friends	0.728	2.70	1.357	0.680	0.610
F2a_InfoFromDoctor	From doctors or other health professionals	0.673	2.62	1.404	0.639	0.588
**F3 Intention to screen (5 items)**						
C3i_ScreenRegularCare	Routine cancer screening shows that people care for their health and their families	0.845	4.14	1.253	0.748	0.773
C3h_ScreenRegularAll	All people should have regular cancer screenings	0.827	4.08	1.273	0.718	0.751
C3f_ScreenSaveLifes	Cancer screenings help to save lives	0.778	4.20	1.273	0.653	0.646
C3j_PossibleHaveCancerLife	It is possible to get cancer during lifetime	0.628	3.88	1.370	0.493	0.562
C3g_ScreenRegularAge	Friends of my age have cancer screenings regularly	0.618	3.30	1.348	.481	0.479
**F4 Trust in physicians (4 items)**						
P1c_TrustSameCulture	Trust doctors of same cultural background	0.861	3.97	1.011	0.767	0.786
P1b_TrustDifferentRace	Trust doctors of a different race/ethnicity	0.845	3.82	1.085	0.737	0.766
P1a_TrustMedicalAdvise	Trust doctor’s decisions about medical screenings	0.775	3.95	.915	0.653	0.658
P1d_TrustCureDiseases	Trust doctors effectiveness to curing diseases	0.706	3.74	1.035	0.581	0.588
**F5 Preferences about providers (4 items)**						
P2b_SameRace	Preference for a doctor of same race/culture	0.840	2.47	1.403	0.762	0.791
P2a_SameGender	Preference for a doctor of same gender	0.787	2.63	1.430	0.615	0.683
P2c_SameLanguage	Preference for a doctor who speaks same language	0.718	2.82	1.512	0.638	0.692
P2d_SameReligion	Preference for a doctor of same religion	0.698	2.24	1.370	0.571	0.635

^1^ Range 1 to 5. h^2^ = Extraction communality is the proportion of each item’s variance that can be explained by the factor, this is the extent to which an item correlates with all other items in the factor. CITC = Corrected Item-Total correlation (Item discrimination).

**Table 5:** Factors and items in the Barriers Domain.

Factors and Items	Item Description	Factor Loads	Mean ^1^	SD	CITC	h^2^
**F6 Lack awareness (2 items)**						
B1a_TestNoOrdered	Doctor does not order the screening tests	0.809	2.85	1.588	0.610	0.749
B1b_UnsureNeedTest	Being unsure if there is a need for screening	0.801	2.93	1.485	0.610	0.778
**F7 Personal discomfort (5 items)**						
B1d_AfraidTestHarm	Being afraid the test will cause harm if it is not well done	0.799	2.24	1.411	0.704	0.716
B1e_AfraidTestCancer	Being afraid the test will cause cancer	0.758	1.91	1.291	0.647	0.667
B1f_DislikeTestTouching	Dislike having exams in private parts of the body	0.690	2.38	1.469	0.634	0.596
B1c_LackTrustResults	Lack of trust on cancer screening tests	0.682	2.19	1.371	0.606	0.594
B1g_DontWantKnow	Do not want to know if cancer is present	0.658	2.26	1.524	0.580	0.547
**F8 Impediments to screen (6 items)**						
B3b_ForgotMakeAppt	Forgot to make the appointment	0.780	1.87	1.276	0.692	0.689
B3c_ProblemMakeAppt	Had problems making the appointment	0.750	1.74	1.218	0.708	0.689
B3d_DonotKnowWhen	Do not know if it is time to have the exams	0.749	2.16	1.426	0.686	0.659
B3e_NoReminder	Have not received a reminder (mail, call, etc.)	0.698	2.10	1.448	0.666	0.616
B3f_WaitTimeAppt	Waiting too long for an appointment	0.670	1.92	1.279	0.635	0.606
B3a_DontKnowWhere	Do not know where to go for the exam	0.600	2.02	1.457	0.598	0.567
**F9 Lack of resources (5 items)**						
B3k_NoMoney	Lack of money to pay for the exam/copayment	0.774	2.13	1.529	0.699	0.734
B3j_NoInsurance	Lack of health insurance	0.762	2.00	1.517	0.691	0.730
B3i_NoChildCare	Need child/family care services	0.634	1.48	1.053	0.580	0.578
B3h_NoTransportation	Lack of transportation to go to the appointment	0.631	1.60	1.153	0.643	0.621
B3m_NeedCompany	Need company to go to the appointment	0.566	1.68	1.257	0.608	0.585
**F10 Poor English skills (3 items)**						
L1b_ReadEnglish	How well do you read English	−0.915	3.83	1.546	0.961	0.930
L1c_WriteEnglish	How well do you write English	−0.908	3.76	1.618	0.951	0.916
L1a_SpeakEnglish	How well do you speak English	−0.908	3.88	1.518	0.943	0.913
**F11 Communication problems (3 items)**						
L2b_DifficultUnderFast	Hard time understanding doctors who speak quickly	0.763	2.46	1.571	0.652	0.777
L2c_DifficultAccent	Difficulties understanding doctors who have heavy accent	0.713	2.74	1.614	0.276	0.616
L2a_DifficultFillForms	Difficulty to fill out medical forms	0.666	2.19	1.506	0.561	0.734
**F12 Low locus of control (2 items)**						
C4e_DontKnowDecision	Lack knowledge to make own decisions	0.789	3.21	1.580	0.316	0.683
C4d_OffendDoctor	Afraid of offending doctor when making own decisions	0.681	2.63	1.517	0.316	0.595

^1^ Range 1–5. h^2^ = Extraction communality is the proportion of each item’s variance that can be explained by the factor, this is the extent to which an item correlates with all other items in the factor CITC = Corrected Item-Total correlation (Item discrimination).

**Table 6:** Factors and items in the Cultural Domain.

Factors and Items	Item Description	Factor Loads	Mean ^1^	SD	CITC	h^2^
**F13 Stigmas about cancer (2 items)**						
C1b_CancerBadLuck	Cancer is due to bad luck	0.803	1.55	1.055	0.608	0.765
C1c_CancerPunishment	Cancer is a punishment from God	0.799	1.41	0.992	0.608	0.767
**F14 Fatalistic attitude (3 items)**						
C1e_CancerEqualDeath	Cancer is a deadly disease	0.829	3.53	1.519	0.591	0.722
C1f_CancerThinkDeath	When thinking of cancer, automatically think of death	0.798	3.11	1.512	0.614	0.705
C1d_CancerWorstThing	Cancer is the worst thing that can happen to a person	0.730	2.84	1.596	0.528	0.629
**F15 Beliefs about treatment (2 items)**						
C2c_CureGodWill	Cancer can be cured only if it is God’s will	0.736	2.78	1.665	0.354	0.654
C2b_TreatSurgerySpread	Treating cancer with surgery can cause it to spread throughout the body	0.661	2.63	1.395	0.354	0.579
**F16 Beliefs about prevention (5 items)**						
C3b_NoScreenAllDie	No need of cancer screening because everyone dies anyway	0.798	1.64	1.192	0.651	0.728
C3c_NoScreenNothingToDo	No need of cancer screening because you will get it no matter what you do	0.774	1.65	1.182	0.670	0.713
C3a_NoScreenGodHands	No need of cancer screening because it is in God’s hands anyway	0.714	1.70	1.262	0.584	0.614
C3e_NothingToDo	There is no so much people can do to lower chances of getting cancer	0.571	2.23	1.356	0.468	0.446
C3d_NoClearWhatToDo	Too many recommendations about preventing cancer make difficult to choose	0.531	2.66	1.422	0.415	0.486
**F17 Self-determination (4 items)**						
C4f_PreferKnowOptions	Prefer having choices instead of doctor making decisions for me	0.717	3.49	1.502	0.436	0.585
C4g_OkNoFollowAdvice	There are good reasons not to follow the advice of a doctor	0.665	2.75	1.418	0.289	0.550
C4a_OkSelfExamination	Being comfortable checking own body for signs of health problems	0.573	3.67	1.472	0.323	0.465
C4c_SecondOpinion	Prefer a second opinion about health conditions and treatment options	0.524	4.33	1.107	0.382	0.532
**F18 Perceived cancer risk (4 items)**						
R1d_RiskStomach	Perceive risk of stomach cancer	0.895	2.75	0.967	0.833	0.842
R1e_RiskLiver	Perceive risk of liver cancer	0.891	2.72	0.990	0.828	0.834
R1c_RiskColon	Perceive risk of colon cancer	0.856	2.83	1.006	0.774	0.770
R1f_RiskSkin	Perceive risk of skin cancer	0.780	2.77	0.997	0.679	0.658
**F19 Worriedness about cancer (4 items)**						
R2d_WorryStomach	Being worried about having stomach cancer	0.884	1.91	1.197	0.855	0.857
R2e_WorryLiver	Being worried about having liver cancer	0.882	1.87	1.188	0.858	0.854
R2c_WorryColon	Being worried about having colon cancer	0.853	2.07	1.235	0.810	0.800
R2f_WorrySkin	Being worried about having skin cancer	0.838	2.01	1.207	0.755	0.760
**F20 Symptomatic deterrents (4 items)**						
B2c_NoNeedNoSymptoms	No need to have cancer screenings if there are not symptoms	0.836	2.68	1.594	0.807	0.827
B2b_NoNeedNormalResults	No need to have cancer screenings after having several normal screening test results	0.784	2.82	1.573	0.673	0.687
B2a_NoNeedFeelHealthy	No need to have cancer screenings when feeling healthy	0.773	2.45	1.592	0.740	0.743
B2d_NoNeedAge	No need to have cancer screenings when being too young or too old	0.697	2.13	1.421	0.581	0.631

^1^ Range 1–5. h^2^ = Extraction communality is the proportion of each item’s variance that can be explained by the factor, this is the extent to which an item correlates with all other items in the factor. CITC = Corrected Item-Total correlation (Item discrimination).
